# Intravenous Dexmedetomidine as an Adjunct to Neuraxial Anesthesia in Cesarean Delivery: A Retrospective Chart Review

**DOI:** 10.1155/2021/9887825

**Published:** 2021-12-27

**Authors:** Paul R. Davis, Hans P. Sviggum, Daniel J. Delaney, Katherine W. Arendt, Adam K. Jacob, Emily E. Sharpe

**Affiliations:** Department of Anesthesiology and Perioperative Medicine, Mayo Clinic, 200 First St SW, Rochester 55902, MN, USA

## Abstract

**Background:**

Dexmedetomidine is a selective *α*-2 agonist commonly used for sedation that has been used in obstetric anesthesia for multimodal labor analgesia, postcesarean delivery analgesia, and perioperative shivering. This study evaluated the role of intravenous dexmedetomidine to provide rescue analgesia and/or sedation during cesarean delivery under neuraxial anesthesia.

**Methods:**

We conducted a single-center, retrospective cohort study of all parturients undergoing cesarean delivery under neuraxial anesthesia between December 1, 2018, and November 30, 2019, who required supplemental analgesia during the procedure. Patients were divided into two groups: patients who received intravenous dexmedetomidine (Dexmed group) and patients who received adjunct medications such as fentanyl, midazolam, ketamine, and nitrous oxide (Standard group). Primary outcome was incidence of conversion to general anesthesia.

**Results:**

During the study period, 107 patients received adjunct medications. There was no difference in conversion to general anesthesia between the Dexmed group and the Standard group (6% (4/62) vs. 9% (4/45); *p*=0.718). In the Dexmed group, the mean dexmedetomidine dose received was 37 *μ*g (range 10 to 140 *μ*g). While the use of inotropic/vasopressor medications was common and similar in both groups, there was an increase in the incidence of bradycardia (Dexmed 15% vs. Standard 2%; *p*=0.042) but not hypotension (Dexmed 24% vs. Standard 24%; *p*=1.00) in the Dexmed group.

**Conclusion:**

In patients who required supplemental analgesia for cesarean delivery, those who received dexmedetomidine versus other medications had a similar rate of conversion to general anesthesia, a statistically significant increase in bradycardia, but no difference in the incidence of hypotension.

## 1. Introduction

Neuraxial anesthesia is the preferred anesthetic for most patients undergoing cesarean delivery (CD). Intrathecal and epidural anesthesia allow the mother to be awake for the birth of her child, minimize anesthetic exposure to the fetus, and avoid general anesthesia, which is associated with greater maternal anesthesia-related adverse events [1]. At times, neuraxial anesthesia is not sufficient for patient comfort throughout the entire surgical procedure. Maternal emotional distress and pain during surgery are leading causes of litigation during CD, and prompt recognition and treatment of inadequate neuraxial blockade are important [2].

A 5-year audit of 5080 cesarean deliveries at a United Kingdom hospital described a rate of conversion from regional anesthesia to general anesthesia of 0.8% for elective and 4.9% for emergent procedures [3]. The percentage of patients who did not achieve a completely pain-free procedure with a regional technique was 6% for spinal, 24% for epidurals, and 18% for combined spinal-epidurals [3]. When inadequate anesthesia occurs, patients benefit from additional analgesic or anesthetic options. Repeat neuraxial procedures or conversion to general anesthesia are options for failed neuraxial anesthesia; however, sedation is often utilized to supplement a neuraxial block after the procedure has begun. Commonly used sedation agents include fentanyl, midazolam, nitrous oxide, ketamine, or propofol, but risks from these agents include but are not limited to apnea, hallucination, and impaired memory formation.

Intravenous (IV) dexmedetomidine is commonly used for sedation in the nonpregnant population through a site of action in the subcortical system that mimics natural sleep [4]. As an active D-isomer of medetomidine, it is a highly selective *α*-2 agonist and is similar to clonidine [5]. It has been utilized for sedation for awake fiberoptic intubations [6, 7] and awake craniotomies in pregnancy [8]. In addition to sedation, dexmedetomidine demonstrates analgesic and antisympathetic effects. It has been reported to provide safe labor analgesia in parturients who are unable to have neuraxial analgesia [9–12], and has an opioid-sparing effect during labor [13]. It can extend spinal anesthesia by 34% (*p* < 0.00001) [14], and its use has resulted in better pain scores, higher analgesic satisfaction, and less rescue analgesia in the postpartum period [15].

How dexmedetomidine enhances local anesthetic action is not well understood but is possibly related to local vasoconstriction or binding to *α*-2 receptors on nerves. Dexmedetomidine has also been shown to be a relatively safe medication, with hypotension and bradycardia as the primary side effects [16], and is thought to have minimal fetal transfer or physiological effects during labor [17–19]. A meta-analysis evaluating the fetal effects of dexmedetomidine administered at induction or as part of the neuraxial anesthetic for CD found no significant effect on Apgar scores or umbilical blood gas values [20].

The use of dexmedetomidine in the ICU and operating room continues to become more common, but there is a paucity of data regarding the use of IV dexmedetomidine as a method to provide sedation to supplement neuraxial anesthesia during CD. With its successful history of use outside of the obstetric practice, its effectiveness to safely sedate and provide additional analgesia with minimal respiratory effects during a CD is encouraging. In this single-center, retrospective study, we report the use of IV dexmedetomidine as a supplement to neuraxial anesthesia during CD and describe the incidence of conversion to general anesthesia in parturients receiving IV dexmedetomidine compared to other sedative medications.

## 2. Methods

This study was a single-center, retrospective cohort study of all parturients undergoing cesarean delivery under neuraxial anesthesia between December 1, 2018, and November 30, 2019, who required supplemental analgesia during the procedure. Patients were divided into two groups: patients who received intravenous dexmedetomidine (Dexmed group) and patients who received adjunct medications such as fentanyl, midazolam, ketamine, and nitrous oxide (Standard group). The Mayo Clinic Institutional Review Board in Rochester, Minnesota approved this study, and this manuscript adheres to the STROBE guidelines. The sample size was determined by convenience sampling of one year of patients having cesarean delivery at our institution. Patients were included in the study if they had a CD, had neuraxial anesthesia, and required supplemental medications such as dexmedetomidine, fentanyl, midazolam, ketamine, nitrous oxide, or propofol. Patients were excluded if they had general anesthesia, an intrauterine fetal demise, or refused research authorization. The primary outcome was the incidence of conversion to general anesthesia. Secondary outcomes included incidence and duration of hypotension, bradycardia, oxygen desaturation, dexmedetomidine usage patterns, inotropic/vasopressor use, and antiemetic use.

The medical records were manually reviewed. Patient and obstetric characteristics including age, race, body mass index (BMI) at time of delivery, gestational age, gravidity, parity (including the current delivery), and number of previous CDs were recorded. Procedural information was collected, including indication for CD, length of procedure, primary anesthetic, and emergency status. Details about adjunct medications including type of medication, dose, and timing were collected.

The neuraxial anesthesia techniques included spinal, epidural, dural puncture epidural (DPE), or combined spinal epidural (CSE). Patients who received a spinal or CSE typically received an intrathecal dose of 12 to 15 mg of 0.75% bupivacaine in 8.25% dextrose with fentanyl 15 mcg and morphine 150 mcg. In addition, a phenylephrine infusion is started in all patients who receive a spinal anesthetic. Patients who received an epidural were typically initially loaded with either 15 to 20 mL of 2% lidocaine with 1 : 200,000 epinephrine and bicarbonate or 15–20 mL of 3-chloroprocaine, in addition to fentanyl 100 mcg and morphine 2 mg. Those who had either an epidural or CSE and required further dosing intraoperatively typically received 2% lidocaine with 1 : 200,000 epinephrine in 5 mL aliquots. Neuraxial blocks were most commonly placed by either a senior resident or fellow, with staff placing the neuraxial block when residents or fellows were unable to.

Each patient's vital signs (including heart rate, blood pressure, and oxygen saturation) were reviewed starting with the initiation of supplemental medications, and abnormalities were noted. All patients were monitored using continuous pulse oximetry, continuous electrocardiogram monitoring, and a blood pressure recording every three minutes, and our electronic medical record system was able to record these at 1-minute intervals for retrospective review. Specifically, bradycardia was defined as a heart rate less than 50 beats per minute (bpm), hypotension was defined as a systolic blood pressure (SBP) < 90 mmHg or a mean arterial pressure (MAP) < 60 mmHg, and oxygen desaturation was defined as an oxygen saturation (SpO_2_) <90%. The duration of each vital sign abnormality was also recorded.

Medical records were manually reviewed and data was recorded in REDCap version 9.1.15 software (Vanderbilt University). The statistical analysis was completed by using JMP version 14.1.0 software (SAS Institute Inc.). All continuous variables were summarized as a median with the first and third quartiles listed, and they were compared using the Wilcoxon rank-sum test. All categorical variables were summarized as a number and percentage and were compared using the Fisher's exact test. “Durations” of events and “Time to First Event” are summarized only for those patients experiencing the given event. Significance for all comparisons was defined as a *p* value <0.05.

## 3. Results

**Figure 1:**
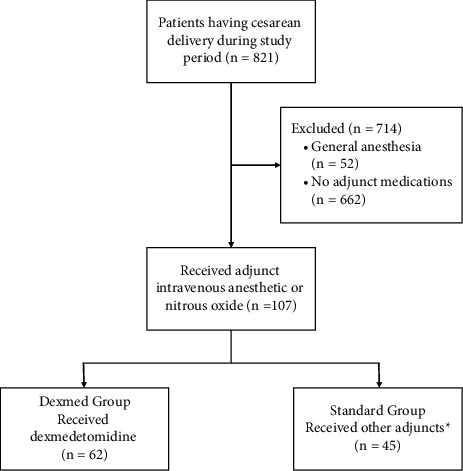
Flowsheet of patient inclusion and exclusion. Flow diagram of patient selection. ^*∗*^Fentanyl, midazolam, ketamine, nitrous oxide, propofol, or a combination of these medications.

During the study period, 821 patients had a CD, and 107 patients received adjunct IV anesthetics or nitrous oxide (Figure 1). A total of 62 (57.9%) women received dexmedetomidine alone or in combination with other medications (Dexmed group) and 45 (42.1%) women received fentanyl, midazolam, ketamine, nitrous oxide, propofol, or a combination of these medications (Standard group). Patient demographics and obstetric information are summarized in Table 1. There was no difference in patient characteristics between the two groups. There were no differences in the duration of the procedure, type of anesthesia, or emergency status between groups (Table 1). The most common indications for CD were failed vaginal delivery and previous CD. The majority of patients in both groups were having their first CD. There was no difference in conversion to general anesthesia between groups (4/62; 6% vs. 4/45; 9%, *p*=0.718).

**Figure 2:**
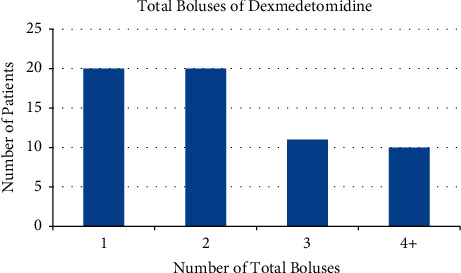
Number of dexmedetomidine boluses administered. Bar graph of the number of patients who received a given number of dexmedetomidine boluses.

Most patients who received dexmedetomidine received 1 or 2 boluses of the medication with a total mean dose of 37 *μ*g (range: 10 *μ*g to 140 *μ*g) (Figure 2). In the Dexmed group, 32 patients received dexmedetomidine alone (51.6%), 22 patients received fentanyl (35.5%), 8 received ketamine (12.9%), 5 received nitrous oxide (8.1%), 3 received midazolam (4.8%), and 3 received propofol (4.8%). In the Standard group, 38 received fentanyl (84.4%), 5 received nitrous oxide (11.1%), 4 received ketamine (8.9%), 2 received midazolam (4.4%), and 2 received propofol (4.4%).

Women who had a spinal anesthetic received adjunct medications later in the surgical procedure compared to women with a preexisting labor epidural (median (IQR) duration of 61 (39,83) min after incision vs. 46 (26, 59) min, respectively; *p*=0.001). This difference was consistent between women in the Dexmed group (spinal 55 (35.5, 72) min vs. epidural 33 (23.5, 49.5) min; *p*=0.003) but not for women in the Standard group (spinal 73 (44.25, 92.25) min vs. epidural 56.5 (42.25, 67.5) min; *p*=0.115). One patient in the Standard group was excluded from this analysis as the time of neuraxial medication administration was missing from the chart. In the Standard group, 7% (3/45) of patients received at least one adjunct prior to delivery, and 11% (7/62) of patients in the Dexmed group received dexmedetomidine prior to delivery.

The incidence and duration of vital sign changes after adjunct medications is described in Table 2. There was no statistically significant difference in the incidence of hypotension, but the Dexmed group displayed an increase in the duration of hypotension when it did occur (6 min vs. 3 min; *p*=0.013). There was also a statistically significant greater incidence of bradycardia in parturients in the Dexmed group compared to the Standard group (9 (15%) vs. 1 (2%); *p*=0.042). Episodes of oxygen desaturation were rare and of short duration in both groups.

There was no statistically significant difference in inotropic or vasopressor medication use between the two groups (Table 3). In total, 90% (56/62) of the Dexmed group received an inotropic or vasopressor medication compared to 91% (41/45) of the Standard group. Additionally, there was no difference in the timing of vasopressor administration in relation to adjunct medication administration.

There was no difference in antiemetic use between the groups. The majority of patients in both groups received ondansetron (58 (94%) in the Dexmed group and 43 (96%) in the Standard group; *p*=1.000) and dexamethasone (43 (69%) in the Dexmed group and 28 (62%) in the Standard group; *p*=0.535). Droperidol was used less frequently (10 (16%) parturients in the Dexmed group and 2 (4%) in the Standard group (*p*=0.069)).

## 4. Discussion

Dexmedetomidine shows promise as a viable option for intraoperative rescue analgesia during CD. In this retrospective study, parturients in the Dexmed and the Standard groups had similar rates of conversion to general anesthesia for an inadequate neuraxial block with minimally different side effect profiles. Dexmedetomidine continues to have an increasing role in obstetrical multimodal pain management, but current dosing studies have indicated a wide spectrum of dosages and dosing strategies [21, 22].

Obstetric anesthesia experts encourage neuraxial anesthesia as the preferred mode of anesthesia for CD. Recently, the Society of Obstetric Anesthesia and Perinatology created the Center of Excellence designation to recognize institutions that demonstrate excellence in obstetric anesthesia care and to set a national benchmark level of expected care for parturients. Among the listed criteria is the recommendation that institutions have a general anesthesia rate for CD of 5% or less [23].

To avoid conversion to general anesthesia because of inadequate neuraxial blockade, systemic adjuncts such as IV anesthetics/analgesics or inhaled nitrous oxide are often given but rarely reported. The incidence of intraoperative pain during cesarean delivery has not been well studied, but Keltz et al. found 11.9% of parturients undergoing spinal anesthesia for elective CD reported intraoperative pain [24]. Even more concerning is that anesthesiologists and obstetricians were unable to accurately identify a patient's pain during surgery in this study population [24]. Clevenger et al. reported that 17.8% of parturients undergoing a CD under regional anesthesia received at least one systemic adjunct [25]. This rate is similar to the findings of our study where 17.2% of parturients having a CD under regional anesthesia received an adjunct medication. Interestingly, a previous study found that activation of a labor epidural was associated with a higher incidence of anesthetic adjuncts compared to de novo spinal anesthesia [25]. This makes intuitive sense, as spinal anesthesia has been shown to produce a superior quality of anesthesia compared to epidural [26].

Dosing for IV dexmedetomidine remains variable, and in our study, the mean total dose of dexmedetomidine administered was 37 *μ*g. Xiong et al. performed a modified two-stage Dixon up-and-down sequential dose-finding study for the ED50 and ED95 of an IV dexmedetomidine loading dose (given over 15 min) for procedural sedation. The ED50 was 1.58 *μ*g/kg and the ED95 was 1.80 *μ*g/kg in pregnant women having CD which was higher than the ED50 and ED95 for nonpregnant patients having a gynecologic procedure (0.96 *μ*g/kg and 1.1 *μ*g/kg, respectively) [21]. A subsequent study found the ED50 and ED95 of loading IV dexmedetomidine according to a Dixon up-and-down methodology to be 0.82 *μ*g/kg and 0.96 *μ*g/kg respectively, in order to obtain a Ramsay sedation scale of ≥3 (drowsy but responds to command) [22]. Both of these studies targeted a higher level of sedation than in our study but demonstrated the range of safe and effective dexmedetomidine dosing strategies.

Dexmedetomidine was commonly administered with fentanyl in our study. Because *α*-2 agonists have demonstrated a synergistic effect with fentanyl, dexmedetomidine and opioids may provide more effective adjunctive analgesia in CD than either agent alone [27]. Patients in the Dexmed and Standard groups had similar rates of conversion to general anesthesia, but as the sample size was determined by a one-year convenience sampling, it was underpowered to detect a statistically different conversion rate to general anesthesia given the observed incidence rate. Future prospective randomized controlled trials should evaluate if dexmedetomidine alone, dexmedetomidine plus fentanyl, fentanyl alone, or another adjuvant agent is most effective in treating pain or anxiety during CD and avoiding conversion to general anesthesia.

Primary side effects of dexmedetomidine include bradycardia and hypotension; therefore, close monitoring of the patient's vital signs is important [16]. In this study, there was an increased incidence of bradycardia for the Dexmed group, but there was no difference in the incidence of hypotension or in inotropic or vasopressor medication use. It is commonly taught that dexmedetomidine does not cause respiratory depression [28]; however, a recent study by Lodenius et al. has called that into question reporting airway collapsibility and reductions in ventilatory drive similar to propofol during infusions [29]. Minimal issues with oxygen desaturation were observed in our patient population.

Dexmedetomidine has been useful in reducing the incidence of postoperative nausea and vomiting (NNT = 9.3) [16]. In this study, we sought to examine the effect of dexmedetomidine on nausea and vomiting by recording the use of antiemetic medications in the two groups. The majority of patients in both groups received ondansetron and dexamethasone. Droperidol is commonly given as a third-line agent at our facility and was used minimally in both groups. There was no difference in antiemetic administration between the Dexmed and Standard groups.

Our study has the inherent limitations of a retrospective chart review, including possible charting omissions and uncertainty in the causes of various management decisions, such as the specific indication for each administered medication. In addition, the patient groups were heterogenous with women receiving both spinal and epidural anesthesia for cesarean delivery. There may have been a selection bias regarding the patients who received dexmedetomidine compared to those in the Standard group. Furthermore, the dose of dexmedetomidine was not standardized. We were unable to assess pain scores during CD and patient satisfaction which is important for patient outcomes and experience during inadequate neuraxial blockade. The low incidence of an adverse outcome (such as hypotension or bradycardia) in a small dataset may underestimate the real risk when larger populations are exposed to this treatment. Furthermore, this study is likely underpowered to find a difference in certain outcomes that could still be clinically important. Finally, this study was performed at a single institution and may not be generalizable to other institutions.

In conclusion, this study reports the use of dexmedetomidine as an adjunct medication for inadequate neuraxial anesthesia during CD. While there was a trend toward lower rates of conversion to general anesthesia in parturients who received dexmedetomidine (6% vs. 9%, *p*=0.718), ultimately this trend was nonsignificant. This study also showed a small increase in bradycardia in parturients who received dexmedetomidine compared to patients in the Standard group. The use of dexmedetomidine to supplement neuraxial anesthesia is an important addition to the number of previously reported off-label applications of dexmedetomidine in obstetric anesthesiology. Continuing to gain a better understanding of the ideal role for this medication in parturients requires further investigation. Future prospective randomized studies should be completed to evaluate whether dexmedetomidine in conjunction with and in comparison to other analgesics, anxiolytics, or anesthetics impacts conversion to general anesthesia, patient pain, and ultimately patient satisfaction when neuraxial anesthesia for CD is suboptimal.

## Figures and Tables

**Table 1:** Patient demographics and surgical characteristics.

	Dexmedetomidine	Others	*p* value
Total number of patients	62	45	
Age^*∗*^ (years)	31 (29, 34)	32 (29, 36)	0.22
Gestational age^*∗*^	39 (37, 39)	39 (36, 39)	0.58
*Race* ^∗∗^			
Caucasian	54 (87%)	35 (78%)	0.3
African American	2 (3%)	3 (7%)	0.65
Hispanic	1 (2%)	1 (2%)	1.00
Asian/Pacific Islander	3 (5%)	2 (4%)	1.00
African	1 (2%)	2 (4%)	0.57
Others	1 (2%)	2 (4%)	0.57
Body mass index	34.2 (28.0, 40.0)	32.6 (28.1, 39.0)	0.67
Gravidity^*∗*^	2 (1, 3)	2 (2, 4)	0.82
Parity^*∗*^	2 (1, 2)	2 (1, 3)	0.42
*Indication for cesarean delivery* ^∗∗^			
Elective	2 (3%)	3 (7%)	0.65
Repeat	19 (31%)	14 (31%)	1.00
Malpresentation	4 (6%)	4 (9%)	0.72
Failure of vaginal delivery	21 (34%)	12 (27%)	0.53
NRFHT	8 (13%)	10 (22%)	0.30
Others	8 (13%)	2 (4%)	0.19
*Number of previous cesarean deliveries* ^∗∗^			
Primary	37 (60%)	23 (51%)	0.43
Repeat	25 (40%)	22 (49%)	0.43
Length of surgery (min)^*∗*^	71 (53, 86)	70 (58, 89)	0.44
*Type of anesthesia* ^∗∗^			
Spinal	33 (53%)	26 (58%)	0.70
Preexisting epidural	29 (47%)	19 (42%)	0.70
*Emergency status* ^∗∗^			
Emergent	35 (56%)	24 (53%)	0.84
Nonemergent	27 (44%)	21 (47%)	0.84
Conversion to general anesthesia^∗∗^	4 (6%)	4 (9%)	0.72

^*∗*^Median (Q1, Q3), Wilcoxon rank-sum test. ^∗∗^*n* (%), Fisher's exact test.

**Table 2:** Incidence of general anesthesia conversion, hypotension, bradycardia, and O_2_ desaturation with dexmedetomidine versus other IV adjuncts or nitrous oxide.

	Dexmedetomidine (*n* = 62)	Others (*n* = 45)	*p* value
Incidence of hypotension^∗∗^	15 (24%)	11 (24%)	1.00
Duration of hypotension (minutes)^*∗*^	6 (3, 27)	3 (3, 6)	0.013
Incidence of bradycardia^∗∗^	9 (15%)	1 (2%)	0.042
Duration of bradycardia (minutes)^*∗*^	2 (1, 2.5)	1	0.35
Incidence of O_2_ desaturation^∗∗^	1 (2%)	0 (0%)	1.00
Duration of O_2_ desaturation (minutes)^*∗*^	2	n/a	n/a

^*∗*^Wilcoxon rank-sum test. ^∗∗^Fisher's exact test. The data are presented as *n* (%) and median (Q1, Q3).

**Table 3:** Inotrope and vasopressor use.

	Dexmedetomidine (*n* = 62)	Others (*n* = 45)	*p* value
Any vasopressor or inotrope	56 (90%)	41 (91%)	1.00
Phenylephrine infusion	45 (73%)	31 (69%)	0.83
Phenylephrine bolus	34 (55%)	28 (62%)	0.55
Phenylephrine bolus as rescue^*∗*^ dose^∗∗^	17 (50%)	10 (36%)	0.31
Ephedrine bolus	12 (19%)	10 (22%)	0.81
Ephedrine bolus as rescue^*∗*^ dose^∗∗^	6 (50%)	3 (30%)	0.41
Glycopyrrolate bolus	3 (5%)	1 (2%)	0.64
Glycopyrrolate bolus as rescue^*∗*^ dose^∗∗^	1 (33%)	0 (0%)	1.00

The data are presented as *n* (%), Fisher's exact test. ^*∗*^Rescue dose is defined as a medication given in response to a change in blood pressure after dexmedetomidine or other adjunct medication has been previously given. ^∗∗^% refers to percent of patients receiving medication from abovementioned row.

## Data Availability

The data that support the findings of this study are available from the corresponding author upon reasonable request.
